# Comparative effectiveness of non-pharmacological interventions on health-related quality of life in ICU patients: a network meta-analysis

**DOI:** 10.3389/fmed.2026.1811907

**Published:** 2026-05-29

**Authors:** Wenhui Yu, Lei Huang, Dan Lv, Meng Li, Yanlin Huang, Yushi Li, Xiaoping Wang

**Affiliations:** 1 Department of Intensive Care Medicine, Tianjin University Central Hospital/Tianjin Third Central Hospital, Tianjin, China; 2 The Third Central Clinical College of Tianjin Medical University, Tianjin, China; 3 Tianjin Key Laboratory of Extracorporeal Life Support for Critical Diseases; Tianjin Artificial Cell Engineering Technology Research Center; Tianjin Institute of Hepatobiliary Disease; Tianjin Institute of Geriatrics; Tianjin Third Central Hospital Branch, Tianjin, China; 4 Department of Heart Center, Tianjin University Central Hospital/Tianjin Third Central Hospital, Tianjin, China; 5 Department of Nursing, Tianjin University Central Hospital/Tianjin Third Central Hospital, Tianjin, China

**Keywords:** critical illness survivorship, health-related outcomes, intensive care unit, network meta-analysis, non-pharmacological intervention, post-ICU recovery, quality of life, rehabilitation strategies

## Abstract

**Background:**

Intensive care unit (ICU) patients often experience long-term physical and psychological impairments that reduce health-related quality of life (HRQoL). Non-pharmacological interventions (NPIs) are increasingly used, but their comparative effectiveness remains uncertain.

**Objective:**

To systematically evaluate NPIs for improving HRQoL in ICU patients using a network meta-analysis (NMA), following PICOS and PRISMA-NMA guidelines.

**Design:**

Systematic review and NMA with a pre-registered protocol (PROSPERO: CRD42024626250).

**Methods:**

We searched PubMed, Embase, Cochrane Library, Web of Science, and EBSCO to November 2024 for randomized controlled trials enrolling adult ICU patients, comparing NPIs with usual care, placebo, or other NPIs. Eligible studies reported validated HRQoL outcomes (SF-36, SF-12, or EQ-5D). Two reviewers independently performed study selection, data extraction, and risk-of-bias assessment. NMA was conducted using RevMan 5.3 and Stata 17.0. Standardized mean differences (SMDs) with 95% confidence intervals (CIs) were calculated, and interventions were ranked by surface under the cumulative ranking curve (SUCRA). Only published, peer-reviewed data were included.

**Results:**

Nineteen RCTs (2,654 patients, 12 NPIs) met inclusion criteria. For overall HRQoL, ICU diary (SMD: −0.30; 95% CI: −0.71–0.10), cognitive therapy plus mobile application (APP+CT) (SMD: −0.26; 95% CI: −0.85–0.33), and electrical muscle stimulation plus physical activity (EMS + PA) (SMD: −0.06; 95% CI: −0.72–0.59) showed the highest SUCRA probabilities. For physical component summary (PCS), the ABCDE bundle (SMD: −0.50; 95% CI: −1.61–0.60) and structured PA (SMD: 0.08; 95% CI: −0.89–1.06) ranked highest. For mental component summary (MCS), the ABCDE bundle (SMD: −0.43; 95% CI: −1.33–0.46) and application-based cognitive therapy (SMD: −0.18; 95% CI: −0.92–0.55) had the greatest probability of benefit. However, none reached statistical significance, as all confidence intervals crossed zero; SUCRA rankings reflect relative probability rather than confirmed effectiveness.

**Conclusion:**

This NMA found no intervention with statistically significant superiority. ICU diary and the ABCDE bundle appear most promising, yet findings must be interpreted cautiously. Further high quality, multicenter RCTs are needed to validate these results and assess long term outcomes.

**Systematic review registration:**

PROSPERO registration number: CRD42024626250. URL: https://www.crd.york.ac.uk/prospero/display_record.php?ID=CRD42024626250.

## 1 Introduction

Patients admitted to the intensive care unit (ICU) often experience complications—including muscle weakness, pain, agitation, and delirium—that persist well beyond discharge. These sequelae, collectively referred to as post-intensive care syndrome (PICS), contribute to sustained impairments in health-related quality of life (HRQoL) for patients and their families (1, 2). Survivors of severe sepsis, acute respiratory distress syndrome (ARDS), and prolonged ICU stays consistently report lower HRQoL scores compared to the general population, with deficits lasting up to five years post-discharge (3, 35). Despite improvements in ICU survival rates, the burden of long-term functional and psychological disability has shifted attention toward optimizing recovery and quality of life. Rehabilitation efforts span multiple phases—from ICU admission to post-discharge recovery—and require tailored interventions across physical, cognitive, and emotional domains (4).

Pharmacological therapies, while commonly employed, are limited by adverse effects such as delirium, dependency, and mood disturbances, as well as high costs and variable efficacy (5). Consequently, non-pharmacological interventions (NPIs)—including physical rehabilitation, psychological support, nutritional strategies, and digital therapeutics—have gained traction as safer, more holistic alternatives (6, 7). Although individual NPIs have demonstrated benefits in select populations, there is currently no consensus on which interventions are most effective or scalable across diverse ICU settings. Pairwise meta analyses have provided valuable insights into single interventions, but they cannot provide a comprehensive comparison across multiple NPIs simultaneously (8, 9). A network meta-analysis (NMA) is superior in this context because it integrates both direct and indirect evidence, allowing estimation of relative effectiveness among all available NPIs within a unified framework (10, 11). This approach is particularly valuable in ICU rehabilitation, where interventions are heterogeneous and head to head trials remain scarce. Accordingly, the present study systematically examines the comparative impact of NPIs on HRQoL in ICU patients, utilizing validated multidimensional outcome measures and rigorous statistical modeling. We provide detailed reporting of NMA methodology—including model structure, handling of multi-arm trials, and assessment of transitivity—to enhance transparency and reproducibility in accordance with PRISMA-NMA guidelines. Furthermore, because feasibility and resource requirements vary widely across ICU settings, our analysis also considers contextual factors that may influence implementation of high-ranking interventions such as ICU diaries and the ABCDE bundle. Our goal is to identify the most promising strategies to support clinical decision-making and inform future research in post-ICU rehabilitation.

## 2 Methods

This review was conducted following the PICOS framework: Population—adult ICU patients; Interventions—any non-pharmacological intervention; Comparators—usual care, placebo, or alternative NPIs; Outcomes—validated HRQoL measures; Study design—randomized controlled trials. All reporting followed PRISMA-NMA guidelines, and no unpublished data were used. The protocol was registered in the PROSPERO database (CRD 42024626250).

### 2.1 Literature search strategy

We systematically searched PubMed, Embase, Cochrane Library, Web of Science and EBSCO to November 2024 using Medical Subject Headings (MeSH), Emtree terms, and free text keywords related to “intensive care unit,” “critical illness,” “rehabilitation,” and “quality of life.” No language restrictions were applied during the search process; however, only studies with full texts available in English were eligible for inclusion. Full search strings are provided in Supplementary Table S1.

### 2.2 Inclusion and exclusion criteria

Eligible studies were randomized controlled trials (RCTs) involving adult patients (≥18 years) admitted to ICUs, regardless of their underlying condition, gender, nationality, race, or treatment modality. ICU settings encompassed general medical, surgical, cardiothoracic, and mixed ICUs. Outcomes were not stratified by ICU type due to limited reporting. Interventions included electrical muscle stimulation (EMS), nutritional support, cognitive therapy, early mobilization, ICU diaries, or combinations thereof. Controls received placebo, usual care, or alternative NPIs. Studies had to report at least one HRQoL outcome using validated instruments (SF-12, SF-36, or EQ-5D). For feasibility of translation and data extraction, only English full-text studies were included.

Studies were excluded if they were duplicate reports or conference abstracts lacking full data, withdrawn publications, or studies failing to report relevant outcomes.

### 2.3 Study screening and data extraction

Initial screening was conducted by removing duplicate records using EndNote X9. Two reviewers (YWH and HYL) independently assessed the titles and abstracts of all retrieved studies based on the predefined inclusion and exclusion criteria. Any disagreements were resolved through discussion, or by consulting a third reviewer when necessary. Data extraction was performed using a standardized spreadsheet in Excel, capturing key information such as study identifiers (including first author, publication year, country, and study design), participant demographics (mean age, sample size, and sex ratio), and detailed descriptions of both experimental and control interventions, including their components, timing, frequency, intensity, and duration. Outcome measures were extracted at both baseline and post-intervention to minimize bias due to baseline differences across studies. Continuous outcomes were recorded as means with standard deviations; when only medians and interquartile ranges were reported, values were converted using established statistical formulas. Categorical outcomes were expressed as event rates. In cases where outcome data were partially inaccessible, ratio-based estimates were calculated using the initial sample size to ensure consistency across studies. All data were extracted independently by the two reviewers (YWH and HYL) to ensure accuracy and reproducibility.

### 2.4 Risk of bias assessment

The methodological quality of the included RCTs was assessed using the Cochrane Handbook for Systematic Reviews of Interventions (version 5.1.0). Two reviewers (YWH and HYL) independently evaluated each study across six domains: random sequence generation, allocation concealment, blinding of participants, blinding of outcome assessors, completeness of outcome data, and selective reporting—could influence pooled estimates. These potential biases were incorporated into our interpretation of the certainty and robustness of the evidence. Each domain was rated as having a low, high, or unclear risk. Studies were classified as high risk if two or more domains were rated high; as low risk if five or more domains were rated low and none were rated high; and as moderate risk if they did not meet either threshold (12). Overall, the predominant limitation was high performance bias due to infeasible blinding, which reduces confidence in subjective outcomes such as HRQoL. This limitation was explicitly considered in the overall quality assessment, as subjective outcomes like HRQoL are particularly vulnerable to inflation. To address this, results were interpreted with caution, acknowledging the potential influence of both performance and detection bias when synthesizing evidence. Visual representations of the risk of bias assessments were generated using RevMan 5.3 software.

### 2.5 Statistical analysis

All statistical analyses were conducted using Stata version 17.0. Analyses were conducted using a frequentist NMA framework. RevMan version 5.3 (Cochrane Collaboration) was used for pairwise meta-analyses, and consistency was ensured by reporting the same version throughout the manuscript. For multi-arm trials, shared control groups were adjusted using appropriate variance-splitting methods to avoid double-counting. Because no Bayesian models were used, convergence diagnostics were not applicable. All procedures adhered to PRISMA-NMA methodological reporting standards.

Effect sizes were first calculated through pairwise meta-analyses, followed by a NMA to compare the relative efficacy of all included non-pharmacological interventions. The geometry of the evidence network was visualized to illustrate the structure and connectivity of treatment comparisons (13). Each node in the network represented a distinct intervention, and the edges between nodes indicated direct comparisons derived from included randomized controlled trials. Edge thickness was proportional to the number of studies contributing to each comparison, reflecting the weight of evidence.

Transitivity was assessed by examining the comparability of study populations (e.g., age, baseline severity, ICU type), intervention characteristics (timing, duration, components), and distribution of potential effect modifiers. Key effect modifiers—including ICU type, patient severity, intervention intensity, and follow-up duration—were broadly comparable across trials, supporting the validity of indirect comparisons.

Heterogeneity across studies was assessed using the *I^2^* statistic. A fixed-effects model was applied when *I^2^* ≤ 50%; otherwise, a random-effects model was used. Because included studies used different HRQoL instruments (SF-36, SF-12, EQ-5D), standardized mean differences (SMDs) were used to enable pooling. However, SMDs may obscure differences in construct coverage and reduce interpretability, introducing residual measurement heterogeneity.

Global and local inconsistencies were evaluated using loop-specific and the node-splitting methods (14). All outcome measures were treated as continuous variables, and SMDs with 95% confidence intervals (CIs) were calculated to assess the significance of treatment effects. A *p*-value of less than 0.05 was considered statistically significant. TSurface under the cumulative ranking curve (SUCRA) values were computed, ranging from 0 to 100%, with higher values indicating greater probability of benefit (15). Funnel plots were constructed for networks with ≥10 studies to assess potential publication bias, with symmetry interpreted as absence of bias. Subgroup analyses were performed based on follow-up duration and outcome types.

## 3 Results

### 3.1 Literature selection and study characteristics

**Figure 1:**
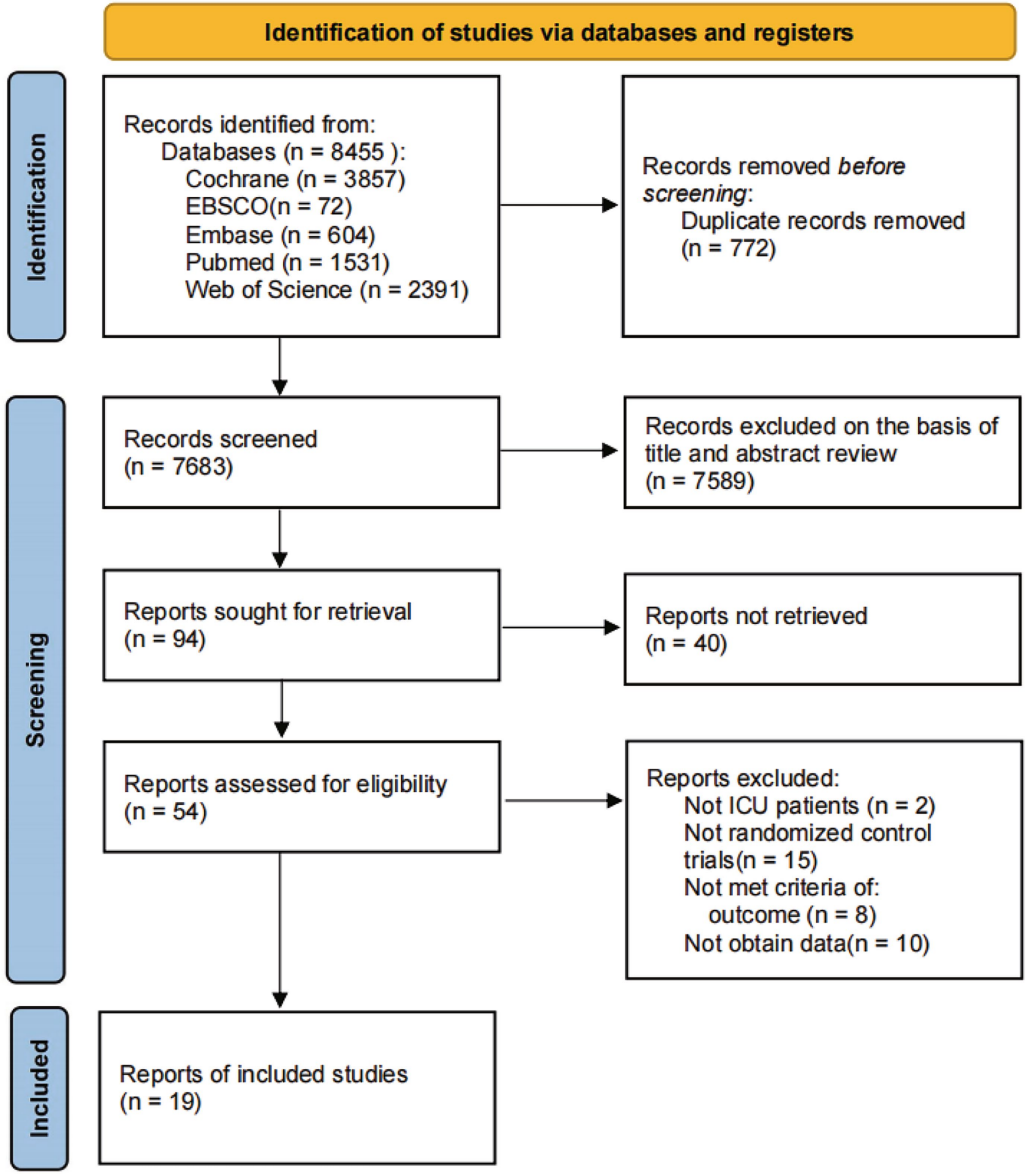
PRISMA flow diagram illustrating the study selection process for the network meta-analysis.

A total of 8,455 records were identified through database searches. After title and abstract screening, 94 studies underwent full-text review. Seventy-five were excluded for not meeting the predefined criteria, resulting in 19 RCTs included in the final analysis (16–34). The study selection process is illustrated in Figure 1.

Supplementary Table S2 summarizes the characteristics of the included studies, published between 2013 and 2024 and enrolling 2,654 ICU patients. Twelve distinct NPIs were evaluated, with several trials incorporating multi-component approaches. Control groups consistently received usual care, routine care, or placebo. Because “usual care” varied across countries, decades, and ICU types, we extracted and summarized its components to improve interpretability and assess potential sources of heterogeneity usual care typically consisted of standard ICU monitoring, physiotherapy as tolerated, nutritional support per local guidelines, routine nursing care, delirium monitoring practices, and psychosocial care. Differences in usual care protocols across settings may have contributed to inconsistency in pooled estimates.

### 3.2 Risk of bias, inconsistency, heterogeneity, and evidence quality assessments

Risk of bias was assessed across seven domains. Random sequence generation was uniformly rated low risk. Allocation concealment was low in 73.7% of studies and unclear in 26.3%. Due to the nature of NPIs, blinding of participants was generally infeasible, resulting in high risk in 84.2% of studies. Blinded outcome assessment was low in 68.4%, unclear in 15.8%, and high in 15.8%. All studies reported complete outcome data. Selective reporting was low in 94.8% and high in 5.2%. Given the high prevalence of performance bias, the overall certainty of evidence is limited and subjective outcomes such as HRQoL may be particularly vulnerable to inflation. Other biases were low in 10.5%, unclear in 84.3%, and high in 5.2%. Overall, the risk of bias was acceptable. Detailed assessments are shown in Supplementary Figure S1.

Global inconsistency testing revealed no significant inconsistency for overall HRQoL, physical component summary (PCS), or mental component summary (MCS) (*p* = 0.3434, 0.8842, and 0.4012, respectively). Local inconsistency testing similarly showed no significant differences between direct and indirect comparisons (all *p* > 0.05).

### 3.3 Primary outcome

#### 3.3.1 Network structure and geometry

**Figure 2:**
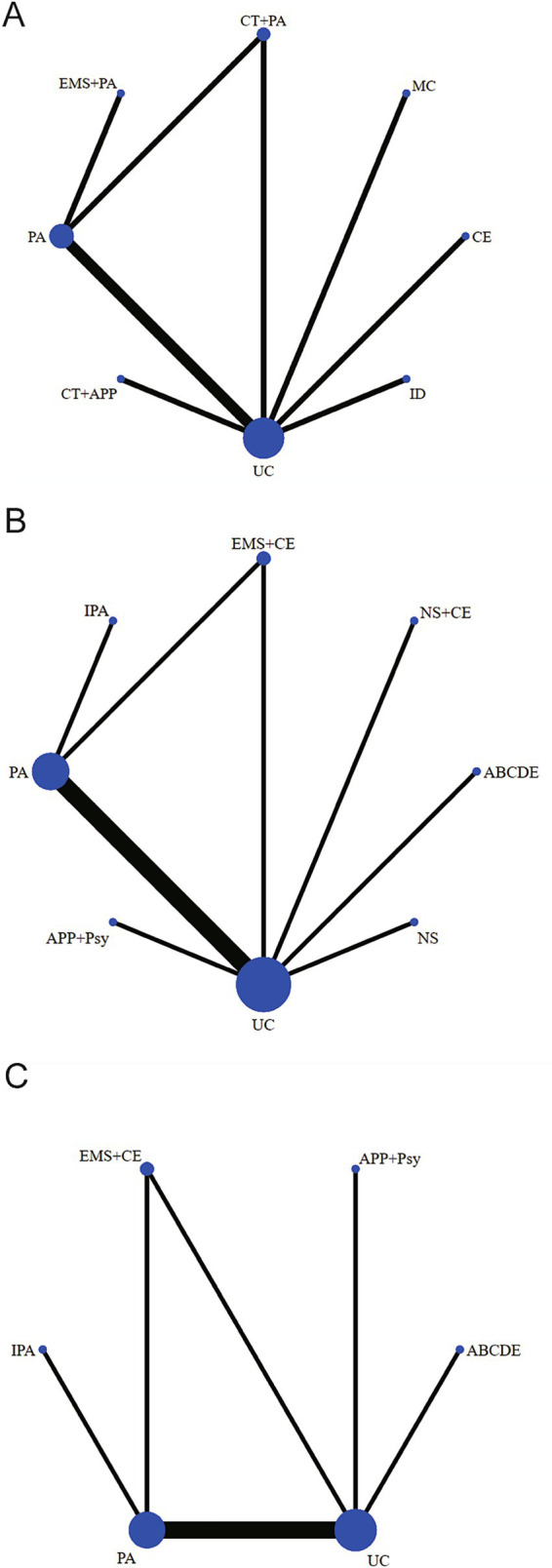
Network plots of non-pharmacological interventions for improving health-related quality of life in ICU patients: **(A)** Overall quality of life, **(B)** Physical component summary (PCS), **(C)** Mental component summary (MCS).

Network plots depicting the relationships among NPIs are shown in Figure 2. The overall HRQoL network contained eight direct or indirect comparisons and one closed loop (Figure 2A). The PCS network included eight direct comparisons and one closed loop (Figure 2B). The MCS network comprised six direct comparisons and one closed loop (Figure 2C).

#### 3.3.2 Rank probabilities

**Figure 3:**
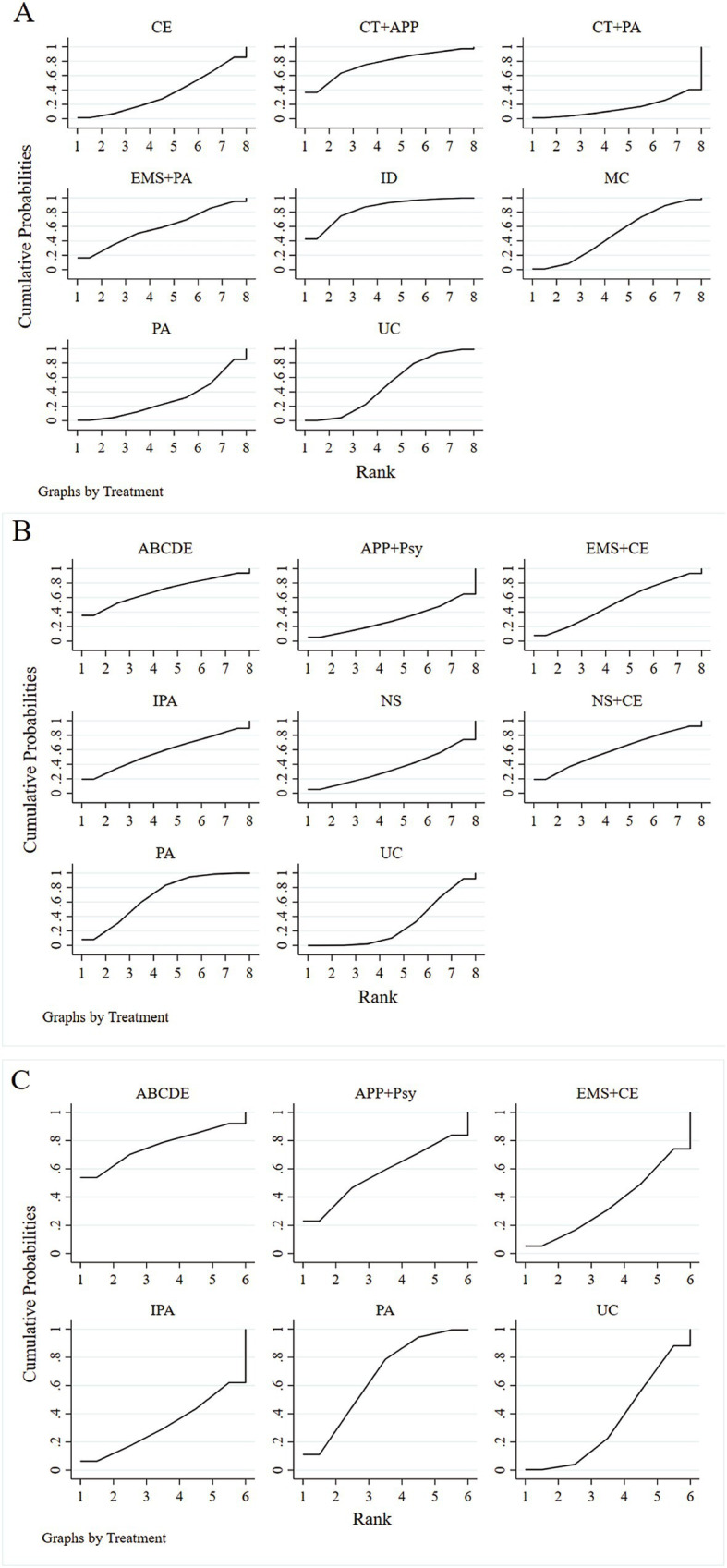
Cumulative surface under the cumulative ranking curve (SUCRA) values for non-pharmacological interventions targeting health-related quality of life in ICU patients: **(A)** Overall quality of life, **(B)** PCS, **(C)** MCS intervention abbreviations: UC, usual care; ABCDE, ABCDE bundle; NS, nutritional support; EMS, electrical muscle stimulation; CT, cognitive therapy; CE, cycle ergometer; ID, ICU diary; PA, physical activity; APP, mobile application; Psy, psychological rehabilitation; IPA, intensive physical activity; MC, multidisciplinary consultations.

Supplementary Figure S2 and Figure 3 illustrate the rank-heat plots and SUCRA distributions, depicting the relative effectiveness of various NPIs in improving HRQoL among ICU patients. Higher SUCRA values indicate greater likelihood of being among the most effective interventions. SUCRA values are presented alongside corresponding effect sizes and 95% CIs to facilitate balanced interpretation. Because all 95% confidence intervals crossed zero, SUCRA rankings should be interpreted as relative probabilities rather than definitive evidence of treatment superiority. Accordingly, even when SUCRA values are high (e.g., for ICU diary or the ABCDE bundle), they should be interpreted with caution if the corresponding confidence intervals are wide and cross the null line, as this indicates uncertainty in the relative probability of effectiveness.

For overall HRQoL (Figure 3A), ICU diary ranked highest (SUCRA 84.7%), followed by cognitive therapy plus mobile application (76.5%), electrical muscle stimulation plus physical activity (58.5%), usual care (50.1%), multidisciplinary consultation (49.9%), cycle ergometer (35.3%), physical activity (29.7%), and cognitive therapy plus physical activity (15.3%). These rankings reflect relative probability only and do not indicate statistically significant differences between interventions.

For PCS (Figure 3B), the ABCDE bundle ranked highest (SUCRA 69.1%), followed by physical activity (67.8%), nutritional support plus cycle ergometer (59.6%), intensive physical activity (57.3%), EMS plus cycle ergometer (51.8%), nutritional support (34.9%), application-based cognitive therapy (APP+CT) (30.4%), and usual care (29.0%). Again, these rankings should be interpreted cautiously because effect sizes were not statistically significant.

For MCS (Figure 3C), the ABCDE bundle again ranked highest (76.3%), followed by physical activity (65.3%), APP+CT (57.8%), EMS + CE (34.5%), usual care (33.4%), and intensive physical activity (32.8%). Although these interventions ranked highly, none demonstrated statistically significant superiority.

#### 3.3.3 Meta-analysis results

**Figure 4:**
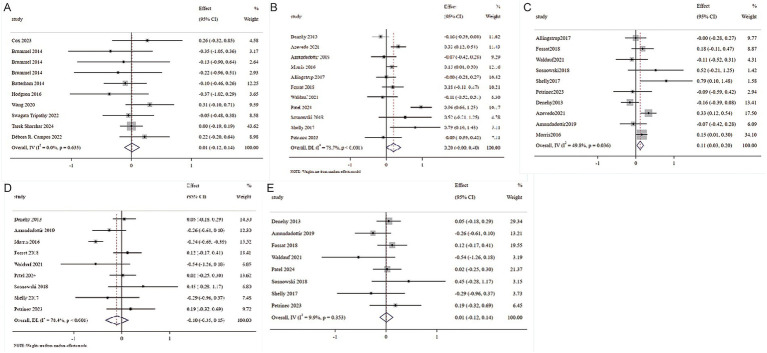
Forest plots of network meta-analysis results. **(A)** Overall quality of life, **(B)** PCS, **(C)** MCS, **(D)** PCS conducted a sensitivity analysis after excluding the literature, **(E)** MCS conducted a sensitivity analysis after excluding the literature.

Forest plots for overall HRQoL, PCS, and MCS are shown in Figure 4. Overall HRQoL demonstrated no heterogeneity (*I^2^* = 0%, *p* > 0.05; Figure 4A). Due to the limited number of studies (n < 3), subgroup analysis was not feasible.

Substantial heterogeneity was observed for PCS (*I^2^* = 78.7%, *p* < 0.05; Figure 4B). Subgroup analysis by follow-up duration (Supplementary Figure S3A) and meta-regression (*p* = 0.840) did not identify significant sources. Sensitivity analysis showed that removing one study reduced heterogeneity below 50%, suggesting that individual trials may have disproportionately influenced pooled estimates (Figure 4C).

Similarly, MCS showed considerable heterogeneity (*I^2^* = 78.4%, *p* < 0.05; Figure 4D). Neither subgroup analysis (Supplementary Figure S3B) nor meta-regression (*p* = 0.930) identified significant sources. Sensitivity analysis reduced heterogeneity to <50% (Figure 4E), again indicating that heterogeneity was driven by specific studies rather than systematic differences.

Sequential sensitivity analyses (Supplementary Figure S4) confirmed the stability of effect estimates.

### 3.4 Publication bias

Funnel plots were largely symmetrical (Supplementary Figure S5), and Egger’s tests (*p* > 0.05) indicated no significant publication bias.

### 3.5 Safety evaluation

No serious adverse events were reported. Minor adverse events occurred in two studies (11.11%) (23, 26), including electrode pad allergy, unplanned extubation, agitation, and transient hypotension. All NPIs were considered safe, with adverse events being mild and manageable. However, because only two studies reported safety outcomes, the evidence base is limited and conclusions regarding comparative safety remain inconclusive. A small proportionality of participants withdrew due to intervention-related adverse reactions, but these instances were rare and did not compromise the integrity of the finding.

## 4 Discussion

To our knowledge, this is the first network meta-analysis to systematically evaluate the comparative effectiveness of currently available NPIs for improving HRQoL in ICU patients. A total of 19 RCTs involving 2,654 participants and 12 distinct intervention strategies were included. Our findings suggest that several NPIs may improve HORQoL, but none of the interventions demonstrated statistically significant superiority because all confidence intervals crossed zero. Therefore, SUCRA rankings should be interpreted as indicators of relative probability rather than definitive evidence of effectiveness, and even high SUCRA values remain uncertain and do not confirm effectiveness.

Current HRQoL assessments in ICU populations predominantly rely on generic measurements such as the 36-item Short Form Health Survey (SF-36) and European Quality of Life 5-Dimensions Questionnaire (EQ-5D) (36, 37). Follow-up durations in the included studies ranged from 1 month to 7 years, with most assessments concentrated at 3-, 6-, and 12-month intervals post-discharge. According to SUCRA ranking, ICU diary, cognitive therapy combined with mobile application (APP+CT), and EMS combined with physical activity (EMS + PA) were the top three interventions for improving overall HRQoL. During ICU stays, patients frequently experience memory gaps, perceptual distortions, and stress-related cognitive changes (38, 39), often accompanied by hallucinations or delusions. ICU diaries, grounded in emotional processing theory, aim to reconstruct factual memory and reduce psychological distress by modifying a maladaptive fear structures (34, 40). A non-randomized Swedish study involving 38 ICU patients demonstrated sustained HRQoL improvement at 6, 12, 24, and 36 months post-discharge following diary use and structured follow-up. However, implementation of ICU diaries requires consistent documentation by nurses or family members, which may be challenging in high-workload or resource-limited ICU environments.

The APP intervention was designed with patient-centered functionality, incorporating cognitive behavioral therapy modules such as mindfulness breathing and guided emotional regulation. These features have been shown to reduce anxiety and depression scores (18, 31). Its multidimensional design—integrating symptom tracking, psychological support, and social connectivity—likely contributed to its high SUCRA ranking. Nevertheless, real-world implementation of digital therapeutics may be hindered by patient adherence, technological accessibility, and the need for clinician training, which vary substantially across ICU settings.

While early mobilization is a cornerstone of ICU rehabilitation, standalone physical rehabilitation therapy often yields suboptimal outcomes. Adjunctive strategies such as nutrition support, massage, and EMS—are frequently required to counteract muscle loss (41). Recent randomized trials and meta-analyses confirm that EMS enhances muscle strength and functional recovery in critically ill patients. EMS combined with early mobilization significantly attenuates quadriceps weakness and improves mobility (41). Meta-analyses further demonstrate systemic benefits, including improved muscle strength scores and potential reductions in ICU length of stay (42, 43). Protocol reviews highlight mechanistic pathways such as improved microcirculation, reduced collagen deposition, and enhanced antioxidant capacity (44). However, its clinical application is constrained by the need for specialized equipment, trained personnel, and individualized parameter optimization.

Early mobilization, although beneficial, is subject to practical limitations. Hemodynamic instability, staffing shortages, and inconsistent protocol adherence can hinder implementation. Moreover, therapeutic outcomes are influenced by variability in exercise parameters—such as intensity, frequency, and baseline patient characteristics—underscoring the need for individualized rehabilitation plans. Emerging evidence indicates that each additional day of ICU hospitalization is associated with a 3–11% decline in muscle strength (45), reinforcing the link between prolonged immobilization and functional deterioration.

Among interventions targeting PCS improvement, the ABCDE bundle, structured physical activity, and nutritional support combined with cycle ergometry demonstrated the highest cumulative efficacy. The ABCDE bundle, while effective, requires daily awakening trials, delirium monitoring, and coordinated activity planning, imposing significant workload demands and necessitating interdisciplinary coordination, which may not be feasible in ICUs with limited staffing or training resources (46).

Physical activity interventions—including resistance training, goal-directed mobilization, and functional exercises—stimulate muscle contraction and protein synthesis, thereby mitigating atrophy and restoring essential physical functions such as standing, ambulation. Tailored physical activity also improves cardiopulmonary function, optimizes hemodynamics and metabolism, and facilitates tissue repair. However, inconsistent adherence to rehabilitation protocols in high-intensity ICU settings may attenuate these benefits, and resource-limited ICUs may lack the personnel or equipment necessary for structured mobilization programs (46).

Nutritional supplementation alone has shown limited efficacy in improving physiological HRQoL outcomes (24), despite being underutilized in ICU patients (47). A synergistic “metabolic-exercise” model combining early nutrition and physical therapy has been proposed to enhance amino acid metabolism and reduce muscle catabolism in patients with acute respiratory failure (48). EMS combined with high-protein nutrition has been shown to preserve quadriceps muscle volume (49). This bidirectional synergy creates a positive feedback loop: nutritional substrates fuel exercise-induced anabolism, while cycle ergometry (active/passive modes) enhances nutrient utilization efficiency (48).

Longitudinal studies have identified temporal patterns in post-ICU depression, with prevalence peaking at 3- and 9-month post-discharge as physical disability worsens (31, 34, 50). Comparative effectiveness analyses suggest the ABCDE bundle offers superior MCS benefits compared to both standalone physical activity and digital mental health interventions. Delirium, a common ICU complication, prolongs mechanical ventilation and hospitalization, and may lead to persistent cognitive deficits that significantly impair HRQoL (51). The 2013 clinical practice guidelines for pain, agitation, and delirium management emphasize non-pharmacological prevention strategies (52), and subsequent studies have confirmed the ABCDE bundle’s efficacy in reducing delirium risk (53).

However, the substantial heterogeneity observed in PCS and MCS outcomes suggests that differences in ICU settings (e.g., general medical vs. cardiothoracic ICUs), intervention intensity, follow-up duration, and HRQoL measurement tools (SF-36, SF-12, EQ-5D) may have influenced effect estimates. Although SMDs enable pooling across heterogeneous instruments, they may obscure differences in construct coverage and reduce interpretability. In addition, variability in usual care across countries and decades likely contributed further to inconsistency. Taken together, these factors limit the certainty of pooled results and underscore the need for cautious interpretation when comparing NPIs across diverse ICU populations. In addition, the high prevalence of performance bias across included trials further reduces confidence in the pooled estimates. Because blinding of participants and personnel was generally infeasible, subjective outcomes such as HRQoL are particularly vulnerable to inflation, and apparent improvements may partly reflect expectation or reporting effects rather than true intervention efficacy.

Digital therapeutics show promise in ameliorating psychological distress (54); however, their broader impact depends on addressing equity and sustainability. Without careful integration, digital tools may inadvertently widen disparities in access and outcomes. Beyond psychological symptoms, core MCS domains such as executive dysfunction and memory impairment remain insufficiently targeted, underscoring the need for dedicated neurocognitive rehabilitation strategies. Physical activity may indirectly alleviate anxiety through endorphin release (55), but it cannot substitute for interventions specifically designed to restore higher order cognitive functions.

Strengths and limitations.

This study has several strengths. First, we conducted a comprehensive and systematic search across five major databases and applied rigorous eligibility criteria focused exclusively on randomized controlled trials, thereby enhancing methodological robustness. Second, validated HRQoL instruments and advanced network meta-analytic techniques were used to integrate both direct and indirect evidence. Third, the study adhered to PRISMA-NMA guidelines and incorporated detailed assessments of transitivity, heterogeneity, and risk of bias, improving transparency and reproducibility. In addition, the reference list was updated to include recent ICU rehabilitation trials published after 2020, strengthening the contemporary relevance of the evidence base. Nevertheless, several limitations should be acknowledged. The number of eligible RCTs was relatively small, and direct head-to-head comparisons between NPIs were sparse, reducing the precision of indirect estimates. HRQoL outcomes were measured using different instruments (SF-36, SF-12, EQ-5D), which may influence the standardized mean difference (SMD) calculations. Although SMDs enable pooling across heterogeneous measures, they may obscure differences in construct coverage and reduce interpretability, thereby introducing measurement heterogeneity. Substantial clinical and methodological heterogeneity—such as differences in ICU settings, intervention intensity, and follow-up duration—may have influenced pooled estimates. The predominance of high performance bias across trials further limits certainty, as subjective outcomes are particularly vulnerable to inflation. Restricting inclusion to English-language publications may also have introduced selection bias. In addition, safety evidence was limited, preventing firm conclusions; future trials should systematically report adverse events to strengthen the evidence base. Taken together, these factors limit the certainty of pooled results and underscore the need for cautious interpretation.

## 5 Conclusion

This network meta-analysis found no intervention with statistically significant superiority. SUCRA values indicate relative likelihood, not definitive superiority. ICU diary and the ABCDE bundle appear most promising, yet findings must be interpreted cautiously as all confidence intervals crossed zero. Future high-quality multicenter RCTs with standardized outcomes and long-term follow-up are essential to validate these findings.

## Data Availability

The original contributions presented in the study are included in the article/Supplementary material, further inquiries can be directed to the corresponding author.
