# Supplementary material for: Comparative effectiveness of non-pharmacological interventions on health-related quality of life in ICU patients: a network meta-analysis
Source: Front Med (Lausanne). 2026 May 29;13:1811907. doi: 10.3389/fmed.2026.1811907 (PMC13260402; doi:10.3389/fmed.2026.1811907)
Supplement: Supplementary file 1 [file Data_Sheet_1.docx]

**Supplementary Contents**

**Table S1.** Search strategy

**Table S2.** Baseline characteristics of included studies

**Table S3.**Relative effect sizes of diﬀerent interventions according to network meta-analysis (Random model)

**Figure S1.** Quality assessment of included studies using the Cochrane Risk of Bias tool

**Figure S2.** Rank‑heat plot of non‑pharmacological interventions across HRQoL domains

**Figure S3.** The result of Subgroup analysis based on the follow-up time.

**Figure S4.** The result of sensitivity analyses

**Figure S5.** Funnel plots of trials included in the network meta-analysis on PCS symptoms.

**Supplementary Table 1.** Search strategy

| 1.PubMed | | |
| --- | --- | --- |
| Search | number Search Details | Results |
| #1 | "intensive care units"[MeSH Terms] OR "intensive care unit"[Title/Abstract] OR "unit, intensive care"[Title/Abstract] OR "ICU Intensive Care Unit"[Title/Abstract] OR "intensive care units"[Title/Abstract] OR "respiratory care units"[Title/Abstract] OR "critical care"[Title/Abstract] OR "critical illness"[Title/Abstract] OR "critical illnesses"[Title/Abstract] OR "critically ill"[Title/Abstract] OR "intensive care"[Title/Abstract] OR ICU[Title/Abstract] OR "intensive care department"[Title/Abstract] OR "high-dependency care unit"[Title/Abstract] | 338356 |
| #2 | "cognitive behavioral therapy"[MeSH Terms] OR "cognitive therap*"[Title/Abstract] OR "behavior therap*"[Title/Abstract] OR "behaviour therap*"[Title/Abstract] OR "behavioral therap*"[Title/Abstract] OR "behavioural therap*"[Title/Abstract] OR "behavior intervention*"[Title/Abstract] OR "behaviour intervention*"[Title/Abstract] OR "behavioral intervention*"[Title/Abstract] OR "behavioural intervention*"[Title/Abstract] OR "multicomponent behavioral therapy"[Title/Abstract] OR CBT [Title/Abstract] OR "stress manage*"[Title/Abstract] OR problem-sol*[Title/Abstract] OR "problem sol*" [Title/Abstract] OR "problem adaptation therapy"[Title/Abstract] OR "acceptance therap*"[Title/Abstract] OR "commitment treatment*"[Title/Abstract] OR "acceptance and commitment therapy"[Title/Abstract] OR psychotherapy [Title/Abstract] | 146371 |
| #3 | mindfulness[MeSH Terms] OR "mind body therapies"[Title/Abstract] OR "relaxation therapy"[Title/Abstract] OR meditation[Title/Abstract] OR mindfulness[Title/Abstract] OR "body mind*"[Title/Abstract] OR "mind body*"[Title/Abstract] OR "mindfulness based stress reduction"[Title/Abstract] OR "mindfulness based*"[Title/Abstract] OR meditat*[Title/Abstract] OR mindful*[Title/Abstract] OR mbsr*[Title/Abstract] OR mbct*[Title/Abstract] | 31443 |
| #4 | "psycho education*"[Title/Abstract] OR "psycho social education"[Title/Abstract] OR "psycho-social education"[Title/Abstract] | 1810 |
| #5 | "relaxation therapy"[MeSH Terms] OR "relaxation therapy"[Title/Abstract] OR "relaxation therapies"[Title/Abstract] OR "abdominal breathing"[Title/Abstract] OR "deep breathing"[Title/Abstract] OR "progressive muscle relaxation"[Title/Abstract] OR music[Title/Abstract] OR musicotherapy[Title/Abstract] OR sing[Title/Abstract] OR rhythm*[Title/Abstract] OR tempo*[Title/Abstract] | 765206 |
| #6 | "art therapy"[MeSH Terms] OR "art-making"[Title/Abstract] OR drawing[Title/Abstract] OR painting[Title/Abstract] OR calligraphy[Title/Abstract] OR sculpture[Title/Abstract] OR collage[Title/Abstract] OR sketch[Title/Abstract] OR craft[Title/Abstract] OR decorate[Title/Abstract] OR ((printed[Title/Abstract] OR printing[MeSH Terms] OR printing[Title/Abstract] OR print*[Title/Abstract] OR printings[Title/Abstract]) AND making[Title/Abstract]) OR clay[Title/Abstract] | 92283 |
| #7 | "reminiscence therapy"[Title/Abstract] OR "life review therapy"[Title/Abstract] OR "life review program"[Title/Abstract] | 446 |
| #8 | "non-drug"[Title/Abstract] OR "non-pharmacological"[Title/Abstract] | 19053 |
| #9 | exercise[MeSH Terms] OR exercise[Title/Abstract] OR training*[Title/Abstract] OR physical∗[Title/Abstract] OR exergam*[Title/Abstract] OR bicycle*[Title/Abstract] or cycl*[Title/Abstract] OR "electrical stimulation"[Title/Abstract] OR rehabilitat*[Title/Abstract] OR "physical therapy modalit*"[Title/Abstract] OR physiotherap*[Title/Abstract] OR kinesiotherap*[Title/Abstract] OR "exercise therap*"[Title/Abstract] OR "physical exertion"[Title/Abstract] OR "early ambulation"[Title/Abstract] OR mobilization[Title/Abstract] OR "muscle weakness"[Title/Abstract] | 3493361 |
| #10 | "family therapy"[Title/Abstract] | 3817 |
| #11 | "technology-based intervention"[Title/Abstract] OR "internet program"[Title/Abstract] OR "virtual reality"[Title/Abstract] OR "virtual reality"[MeSH Terms] | 22953 |
| #12 | vibration[MeSH Terms] OR vibrate*[Title/Abstract] OR vibrating[Title/Abstract] OR vibration*[Title/Abstract] OR vibrator*[Title/Abstract] | 107451 |
| #13 | diaries[Title/Abstract] OR "ICU diaries"[Title/Abstract] OR "intensive care diaries"[Title/Abstract] OR critical care diaries[Title/Abstract] OR diary[Title/Abstract] OR critical care diary[Title/Abstract] OR "intensive care diary"[Title/Abstract] OR "ICU diary"[Title/Abstract] | 31083 |
| #14 | ("randomized controlled trial"[publication type] OR "controlled clinical trial"[publication type] OR randomized[Title/Abstract] OR randomly[Title/Abstract] OR trial[Title/Abstract] OR groups[Title/Abstract]) NOT animals[MeSH Terms] | 795278 |
| #15 | #3 OR #4 OR #5 OR #6 OR #7 OR #8 OR #9 OR #10 OR #11 OR #12 OR #13 OR #14 | 4478866 |
| #16 | #1 AND #15 AND #16 | 1531 |
| 2.Embase | | |
| #1 | 'intensive care units'/exp OR 'intensive care unit':ab,ti OR 'unit, intensive care':ab,ti OR 'ICU Intensive Care Unit':ab,ti OR 'intensive care units':ab,ti OR 'respiratory care units':ab,ti OR 'critical care':ab,ti OR 'critical illness':ab,ti OR 'critical illnesses':ab,ti OR 'critically ill':ab,ti OR 'intensive care':ab,ti OR ICU:ab,ti OR 'intensive care department':ab,ti OR 'high-dependency care unit':ab,ti | 592372 |
| #2 | 'cognitive therapy'/exp OR 'cognitive therap*':ab,ti OR 'behavior therap*':ab,ti OR 'behaviour therap*':ab,ti OR 'behavioral therap*':ab,ti OR 'behavioural therap*':ab,ti OR 'behavior intervention*':ab,ti OR 'behaviour intervention*':ab,ti OR 'behavioral intervention*':ab,ti OR 'behavioural intervention*':ab,ti OR 'multicomponent behavioral therapy':ab,ti OR CBT:ab,ti OR 'stress manage*':ab,ti OR problem-sol*:ab,ti OR 'problem sol*':ab,ti OR 'problem adaptation therapy':ab,ti OR 'acceptance therap*':ab,ti OR 'commitment treatment*':ab,ti OR 'acceptance and commitment therapy':ab,ti | 152760 |
| #3 | 'mindfulness'/exp OR 'mind-body therapies':ab,ti OR 'relaxation therapy':ab,ti OR meditation:ab,ti OR mindfulness:ab,ti OR 'body mind*':ab,ti OR 'mind body*':ab,ti OR 'mindfulness based stress reduction':ab,ti OR 'mindfulness based*':ab,ti OR meditat*:ab,ti OR mindful*:ab,ti OR mbsr*:ab,ti OR mbct*:ab,ti | 42781 |
| #4 | 'psycho education'/exp OR 'psycho education*':ab,ti OR 'psycho social education':ab,ti OR 'psycho-social education':ab,ti | 15739 |
| #5 | 'relaxation training'/exp OR 'relaxation therapy':ab,ti OR 'relaxation therapies':ab,ti OR 'abdominal breathing':ab,ti OR 'deep breathing':ab,ti OR 'progressive muscle relaxation':ab,ti OR music:ab,ti OR musicotherapy:ab,ti OR sing:ab,ti OR rhythm*:ab,ti OR tempo*:ab,ti | 958611 |
| #6 | 'art therapy'/exp OR 'art making':ab,ti OR drawing:ab,ti OR painting:ab,ti OR calligraphy:ab,ti OR sculpture:ab,ti OR collage:ab,ti OR sketch:ab,ti OR craft:ab,ti OR decorate:ab,ti OR 'print making':ab,ti OR 'clay shoveler`s fracture':ab,ti | 84007 |
| #7 | 'reminiscence therapy'/exp OR 'reminiscence therapy':ab,ti OR 'life review therapy'/exp OR 'life review therapy':ab,ti OR 'life review program':ab,ti | 550 |
| #8 | 'non-drug':ab,ti OR 'non-pharmacological':ab,ti | 28115 |
| #9 | 'exercise'/exp OR exercise:ab,ti OR training*:ab,ti OR physical∗:ab,ti OR exergam∗:ab,ti OR bicycle*:ab,ti or cycl*:ab,ti OR 'electrical stimulation':ab,ti OR rehabilitat*:ab,ti OR 'physical therapy modalit*':ab,ti OR physiotherap*:ab,ti OR kinesiotherap*:ab,ti OR 'exercise therapy':ab,ti OR 'exercise therap*':ab,ti OR 'physical exertion':ab,ti OR 'early ambulation':ab,ti OR mobilization:ab,ti OR 'muscle weakness':ab,ti | 4514412 |
| #10 | 'family therapy'/exp OR 'family therapy':ab,ti | 16574 |
| #11 | 'technology-based intervention':ab,ti OR 'internet program':ab,ti OR 'virtual reality':ab,ti OR 'virtual reality'/exp | 37026 |
| #12 | vibration/exp OR vibrate*:ab,ti OR vibrating:ab,ti OR vibration*:ab,ti OR vibrator*:ab,ti | 156474 |
| #13 | diaries:ab,ti OR 'ICU diaries':ab,ti OR 'intensive care diaries':ab,ti OR 'critical care diaries':ab,ti OR diary:ab,ti OR 'critical care diary':ab,ti OR 'intensive care diary':ab,ti OR 'ICU diary':ab,ti | 48696 |
| #14 | ('crossover procedure':de OR 'double-blind procedure':de OR 'randomized controlled trial':de OR 'single-blind procedure':de OR "controlled clinical trial':de OR random*:ab,ti OR trial:ab,ti OR groups:ab,ti OR 'cross NEXT/1 over*':ab,ti OR 'doubl* NEAR/1 blind*':ab,ti OR 'singl* NEAR/1 blind*':ab,ti ) NOT animal/exp | 645620 |
| #15 | #3 OR #4 OR #5 OR #6 OR #7 OR #8 OR #9 OR #10 OR #11 OR #12 OR #13 OR #14 | 5724008 |
| #16 | #1 AND # 15 AND #16 | 604 |
| 3.Cochrane | | |
| #1 | MeSH descriptor: [intensive care units] explode all trees OR (intensive care unit or unit, intensive care or ICU Intensive Care Unit or intensive care units or respiratory care units or critical care or critical illness or critical illnesses or critically ill or intensive care or ICU or intensive care department or high-dependency care unit):ti,ab,kw | 62518 |
| #2 | MeSH descriptor: [quality of life] explode all trees OR (quality of life or influence or life quality or health related quality of life):ti,ab,kw | 256644 |
| #3 | MeSH descriptor: [Cognitive Behavioral Therapy] explode all trees OR (cognitive therap* or behavior therap* or behaviour therap* or behavioral therap* or behavioural therap* or behavior intervention* or behaviour intervention* or behavioral intervention* or behavioural intervention* or multicomponent behavioral therapy):ti,ab,kw | 150267 |
| #4 | MeSH descriptor: [Mindfulness] explode all trees OR (mind-body therapies or relaxation therapy or meditation or mindfulness or body-mind* or mind-body* or mindfulness based stress reduction or mindfulness based* or meditat* or mindful* or MBSR* or MBCT*):ti,ab,kw | 21984 |
| #5 | (psycho-education* or psycho education* or psycho social education or psycho-social education):ti,ab,kw | 2001 |
| #6 | MeSH descriptor: [Relaxation Therapy] explode all trees OR (relaxation therapy or relaxation therapies or abdominal breathing or deep breathing or progressive muscle relaxation):ti,ab,kw | 13044 |
| #7 | MeSH descriptor: [Art Therapy] explode all trees OR (Art-making or drawing or Painting or Calligraphy or Sculpture or Collage or Sketch or Craft or Decorate or Print making or clay):ti,ab,kw | 4548 |
| #8 | (Reminiscence therapy or Life review therapy or Life review program):ti,ab,kw | 11992 |
| #9 | (non-drug or non-pharmacological):ti,ab,kw | 6563 |
| #10 | MeSH descriptor: [Family Therapy] explode all trees OR (family therapy):ti,ab,kw | 17519 |
| #11 | MeSH descriptor: [virtual reality] explode all trees OR (technology-based intervention or internet program or virtual reality):ti,ab,kw | 14446 |
| #12 | MeSH descriptor: [Vibration] explode all trees OR (vibrate* or vibrating or vibration* or vibrator*):ti,ab,kw | 5493 |
| #13 | (diaries or ICU diaries or intensive care diaries or critical care diaries or diary or critical care diary or intensive care diary or ICU diary):ti,ab,kw | 23880 |
| #14 | MeSH descriptor: [Exercise] explode all trees OR (training* or physical* or exergam* or bicycle* or cycl* or electrical stimulation or rehabilitat* or physical therapy modalit* or physiotherap* or kinesiotherap* or exercise therap* or physical exertion or early ambulation or mobilization):ti,ab,kw | 462494 |
| #15 | MeSH descriptor: [Randomized Controlled Trial] explode all trees OR (controlled clinical trial or random* or trial or groups):ti,ab,kw | 1607835 |
| #16 | #3 OR #4 OR #5 OR #6 OR #7 OR #8 OR #9 OR #10 OR #11 OR #12 OR #13 OR #14 | 600963 |
| #17 | #1 AND #2 AND # 15 AND #16 | 3857 |
| 4.Web of science | | |
| #1 | TS = ("intensive care unit" OR "unit, intensive care" OR "ICU Intensive Care Unit" OR "intensive care units" OR "respiratory care units" OR "critical care" OR "critical illness" OR "critical illnesses" OR "critically ill" OR "intensive care" OR ICU OR "intensive care department" OR "high-dependency care unit") | 343242 |
| #2 | TS = ("quality of life" OR influence OR "life quality" OR "health related quality of life") | 5102384 |
| #3 | TS = ("cognitive behavioral therapy" OR "cognitive therap*" OR "behavior therap*" OR "behaviour therap*" OR "behavioral therap*" OR "behavioural therap*" OR "behavior intervention*" OR "behaviour intervention*" OR "behavioral intervention*" OR "behavioural intervention*" OR "multicomponent behavioral therapy" OR CBT OR stress manage* OR problem-sol* OR problem sol* OR problem adaptation therapy OR acceptance therap* OR commitment treatment* OR acceptance and commitment therapy OR psychotherapy ) | [2038875](https://www.webofscience.com/wos/woscc/summary/a3837087-e695-4789-aac0-8ec6bb8fad01-012661f446/relevance/1) |
| #4 | TS = ("mindfulness" OR "mind body therapies" OR "relaxation therapy" OR "Meditation" OR "body mind*" OR "mind body*" OR "mindfulness based stress reduction" OR "mindfulness based*" OR "meditat*" OR "mindful*" OR "mbsr*" OR "mbct*") | [62217](https://www.webofscience.com/wos/woscc/summary/f75e072e-1ebf-4b37-b514-e706f459c648-0126620b22/relevance/1) |
| #5 | TS = ("psycho education*" OR "psycho social education" OR "psycho-social education") | [2386](https://www.webofscience.com/wos/woscc/summary/512e1fac-6d1f-4000-935e-3b39966f11bc-0126621d86/relevance/1) |
| #6 | TS = ("relaxation therap*" OR "abdominal breathing" OR "deep breathing" OR "progressive muscle relaxation" OR "music" OR "musicotherapy" OR "sing" OR "rhythm*" OR "tempo*") | [1619614](https://www.webofscience.com/wos/woscc/summary/dfe06f1f-2e09-434c-8692-3b67499eaec6-012662264b/relevance/1) |
| #7 | TS = ("art therapy" OR "art-making" OR "drawing" OR "painting" OR "calligraphy" OR "sculpture" OR "collage" OR "sketch" OR "craft" OR "decorate" OR "print*" OR "making" OR "clay") | [2280798](https://www.webofscience.com/wos/woscc/summary/9926e6bc-8ade-449f-b1f3-79390b1969d7-012662528e/relevance/1) |
| #8 | TS = ("reminiscence therapy" OR "life review therapy" OR "life review program") | [595](https://www.webofscience.com/wos/woscc/summary/0fa6666b-8d28-4e3c-9672-4fbdf379789a-01266261d1/relevance/1) |
| #9 | TS = ("non-drug" OR "non-pharmacological") | [18209](https://www.webofscience.com/wos/woscc/summary/9e11575f-cfe6-495d-8e99-4b77acbb0012-0126626c77/relevance/1) |
| #10 | TS = (exercise OR training* OR physical∗ OR exergam∗ OR bicycle* or cycl* OR "electrical stimulation" OR rehabilitation OR rehabilitat* OR "physical therapy modalit*" OR physiotherap* OR kinesiotherap* OR "exercise therapy" OR "exercise therap*" OR "physical exertion" OR "early ambulation" OR mobilization OR "muscle weakness") | [7364489](https://www.webofscience.com/wos/woscc/summary/878beb30-0b24-4eea-9cc0-af1a08041026-0126627f28/relevance/1) |
| #11 | TS = ("family therapy") | [9860](https://www.webofscience.com/wos/woscc/summary/3933c63a-d54f-4040-ad05-21011268833a-01266292fc/relevance/1) |
| #12 | TS =("technology-based intervention" OR "internet program" OR "virtual reality") | [76554](https://www.webofscience.com/wos/woscc/summary/c152b767-622e-4bc7-8fac-a4bba9dc6b5b-01266299fa/relevance/1) |
| #13 | TS = (vibration OR vibrate* OR vibrating OR vibrator*) | [464743](https://www.webofscience.com/wos/woscc/summary/a2f286f8-c478-4f40-a552-44200a8dbdb2-012662a2e5/relevance/1) |
| #14 | TS = (diaries OR "ICU diaries" OR "intensive care diaries" OR critical care diaries OR diary OR critical care diary OR "intensive care diary" OR "ICU diary") | [57585](https://www.webofscience.com/wos/woscc/summary/5c3861b3-1b0d-45ff-abe3-acfabed11e7e-012662ad25/relevance/1) |
| #15 | TS = (("randomized controlled trial" OR "controlled clinical trial" OR randomized OR randomly OR trial OR groups) NOT animals) | 8316074 |
| #16 | #3 OR #4 OR #5 OR #6 OR #7 OR #8 OR #9 OR #10 OR #11 OR #12 OR #13 OR #14 | [12828264](https://www.webofscience.com/wos/woscc/summary/543c9ff8-3863-4b1d-b7f9-39dbfc069b3e-012662cc38/relevance/1) |
| #17 | #1 AND #2 AND #15 AND #16 | [2391](https://www.webofscience.com/wos/woscc/summary/0c99d940-1f7c-4cb5-9b45-bc39c87099b5-012662d9cb/relevance/1) |
| 5.EBSCO | | |
| #1 | TX ("intensive care unit" OR "unit, intensive care" OR "ICU Intensive Care Unit" OR "intensive care units" OR "respiratory care units" OR "critical care" OR "critical illness" OR "critical illnesses" OR "critically ill" OR "intensive care" OR ICU OR "intensive care department" OR "high-dependency care unit") | 433 |
| #2 | TX ("quality of life" OR influence OR "life quality" OR "health related quality of life") | 200917 |
| #3 | TX ("cognitive behavioral therapy" OR "cognitive therap*" OR "behavior therap*" OR "behaviour therap*" OR "behavioral therap*" OR "behavioural therap*" OR "behavior intervention*" OR "behaviour intervention*" OR "behavioral intervention*" OR "behavioural intervention*" OR "multicomponent behavioral therapy" OR CBT OR stress manage* OR problem-sol* OR problem sol* OR problem adaptation therapy OR acceptance therap* OR commitment treatment* OR acceptance and commitment therapy OR psychotherapy ) | 96922 |
| #4 | TX ("mindfulness" OR "mind body therapies" OR "relaxation therapy" OR "Meditation" OR "body mind*" OR "mind body*" OR "mindfulness based stress reduction" OR "mindfulness based*" OR "meditat*" OR "mindful*" OR "mbsr*" OR "mbct*") | 4316 |
| #5 | TX ("psycho education*" OR "psycho social education" OR "psycho-social education") | 727 |
| #6 | TX ("relaxation therap*" OR "abdominal breathing" OR "deep breathing" OR "progressive muscle relaxation" OR "music" OR "musicotherapy" OR "sing" OR "rhythm*" OR "tempo*") | 42067 |
| #7 | TX ("art therapy" OR "art-making" OR "drawing" OR "painting" OR "calligraphy" OR "sculpture" OR "collage" OR "sketch" OR "craft" OR "decorate" OR "print*" OR "making" OR "clay") | 1024181 |
| #8 | TX ("reminiscence therapy" OR "life review therapy" OR "life review program") | 14 |
| #9 | TX ("non-drug" OR "non-pharmacological") | 76 |
| #10 | TX (exercise OR training* OR physical* OR exergam* OR bicycle* or cycl* OR "electrical stimulation" OR rehabilitation OR rehabilitat* OR "physical therapy modalit*" OR physiotherap* OR kinesiotherap* OR "exercise therap*" OR "physical exertion" OR "early ambulation" OR mobilization OR "muscle weakness") | 363981 |
| #11 | TX ("family therapy") | 2239 |
| #12 | TX ("technology-based intervention" OR "internet program" OR "virtual reality") | 2381 |
| #13 | TX (vibration OR vibrate* OR vibrating OR vibrator*) | 496 |
| #14 | TX (diaries OR "ICU diaries" OR "intensive care diaries" OR critical care diaries OR diary OR critical care diary OR "intensive care diary" OR "ICU diary") | 4625 |
| #15 | TX (("randomized controlled trial" OR "controlled clinical trial" OR randomized OR randomly OR trial OR groups) NOT animals) | 407061 |
| #16 | #3 OR #4 OR #5 OR #6 OR #7 OR #8 OR #9 OR #10 OR #11 OR #12 OR #13 OR #14 | 1270373 |
| #17 | #1 AND #2AND # 15 AND #16 | 72 |

**Table S2.** Baseline characteristics of included studies in the network meta-analysis.

| Author, year | Country | Male/Famale | | Age | | Intervention:N | Control:N | Intervention duration | Intervention frequency | Intervention cycle | Outcomes | Follow-up time | Adverse event |
| --- | --- | --- | --- | --- | --- | --- | --- | --- | --- | --- | --- | --- | --- |
|  |  | Intervention | Control | Intervention | Control |  |  |  |  |  |  |  |  |
| Denehy  2013^[35]^ | Australia | 43/31 | 52/24 | 61.4±15.9 | 60.1±15.8 | PA:74 | UC:76 | ICU:15mins;Ward:30mins to 60mins;Outpatients:60mins | ICU:once~twice/day;Ward:once~twice/day;Outpatients:twice/week | 8weeks | SF-36 | - | None |
| Azevedo  2021^[17]^ | Brazil | 53/34 | 46/48 | 67.6±17.8 | 65.3±19.7 | NS+CE:87 | UC:94 | F:15mins | twice/day | NS:7days;CE:21 days | SF-36 | - | - |
| Cox  2023^[18]^ | USA | 3/11 | 10/5 | 52.7±11.5 | 46.5±14.1 | CT+APP:14 | UC:15 | - | once/week | 1month | EQ- VAS | - | - |
| Brummel  2014^[36]^ | USA | T1:13/9;T2:28/15 | 8/14 | T1:62(48–67);T2:62(54–69) | 60(51–69) | PA:22;CT＋PA:43 | UC:22 | I:15mins/23mins；E:20mins | I:Once/day；E:twice/day | Until discharge or independently walk more than 200 feet and perform activities of daily living for two days | EQ- VAS | 12weeks |  |
| Batterham  2014^[20]^ | UK | 19/10 | 19/11 | 42.7(18–65) | 40.5(19–60) | CE:29 | UC:30 | 40mins | F:twice/wk;physiotherapist-led supervised sessions per week | 8 weeks | SF-36 | 8~26weeks | - |
| Amundadottir2019^[21]^ | Europe | 19/10 | 14/7 | 62(50–70) | 64 (58–74) | IPA:29 | PA:21 | ≥20 mins | T1:twice/day；T2:Once/day | Until discharge | SF-36v2 | 12months | - |
| Morris  2016^[22]^ | Australia | 66/84 | 68/82 | 55±17 | 58±14 | PA:150 | UC:150 | - | three times/day | Until icu discharge | SF-36 | 6months | - |
| Hodgson  2016^[23]^ | Australia | 21/8 | 9/12 | 64±12 | 53±15 | PA:29 | UC:21 | 30mins(IMS: 1-2 scores),45mins(IMS:4-6),1 hr (IMS: 7-10) | According to the decision of the physical therapist | Until discharge | EQ-VAS | 6months | Control :2 agitation ,2 transient hypotension;Intervention:1 restless |
| Allingstrup  2017^[24]^ | Germany | 65/35 | 59/40 | 63(51–72) | 68(52–75) | NS:100 | UC:99 | - | - | Until tracheal extubation or ICU discharge | SF-36 | 6months | - |
| Wang  2020^[25]^ | China | 29/17 | 30/19 | 53.04±9.53 | 55.61±10.61 | ID:46 | UC:49 | - | - | 1 week to 1 month after ICU discharge | SF-36 | 3months | - |
| Tripathy  2022^[34]^ | India | - | - | 46.9±17.0 | 46.6±17.6 | ID-3:43 | ID-1:41 | - | - | - | EQ-5D-3L | 3.5months | - |
| Fossat  2018^[26]^ | France | 103/55 | 98/56 | 65±13 | 66±15 | EMS+CE:158 | UC:154 | f:15mins；d:50mins | 5days/week | From randomization to ICU discharge | SF-36 | 6months | Intervention: 1 allergic to the electrode pad, 1 unplanned extubation |
| Waldauf  2021^[27]^ | Czech | 53/22 | 57/18 | 59.9±15.1 | 62.3±15.4 | EMS+CE:75 | PA:75 | 90mins/day,adjusted duration | twice/day | Until the 28th day after randomization or ICU discharge | SF-36 | 6months | - |
| Patel  2024^[28]^ | USA | 58/41 | 55/44 | 57.9(42.3–66.8) | 54.5(41.9–64.7) | PA:99 | UC:99 | 25~30mins | Once/day | Until discharge or recovery to the baseline functional level | SF-36 | 12months | - |
| Sosnowski  2018^[29]^ | Australia | 9/6 | 4/11 | 54.9±15.9 | 60.6±11.0 | ABCDE:15 | UC:15 | - | ABC:Once/day;D:NRS every 2 hours,RASS every 4 hours,CAM-ICU every 12 hours.E: three times a day | until ICU discharge | SF-36 | 90days | None |
| Shelly  2017^[30]^ | India | 11/6 | 10/8 | 59(50.5–65) | 53(42.75–56) | PA:17 | UC:18 | 30~40 mins | five times/week | Up to 4 weeks after discharge | SF-36 | 4weeks | - |
| Petrinec  2023^[31]^ | USA | 16/14 | 17/13 | 55.37±13.43 | 58.50±16.01 | APP+Psy:30 | UC:30 | 15 mins | Finish one class every day | Complete all modules | SF-12 | 60days | - |
| Sharshar  2024^[32]^ | Germany | 165/103 | 164/108 | 62.4(51.3–71.7) | 64.7(55.3–75.2) | MC:268 | UC:272 | - | At ICU discharge, 3 months, 6 months and 12 months | Until 12 months after discharge | EQ -5D-5L | 12months | - |
| Campos  2022^[33]^ | Brazil | 24/10 | 26/14 | 42.5±14.9 | 46.7±17.9 | EMS+PA:34 | PA:40 | 60mins | Once/day,5 days/week | until ICU discharge | EQ-5D-3L | - | - |

Abbreviation: N, number; SD, Standard deviation; SF-36, Short form 36 health survey; SF-36v2, Short form 36 health survey vension 2; SF-12, Short form 36 health survey; EQ 5D-5L, Euro Quality of life-5 dimensions and 5 levels questionnaire; EQ-5D-3L, Euro Quality of life-5 dimensions and 3 levels questionnaire; EQ-VAS, Euro Quality Visual. UC, usual care; ABCDE, ABCDE bundle; NS, nutritional support; EMS, electrical muscle stimulation; CT, cognitive therapy; CE, cycle ergometer; ID, ICU diary; PA, physical activity; APP+Psy, application+psychological rehabilitation (APP‑based cognitive therapy); IPA, intensive physical activity; MC, multidisciplinary consultations

**Supplementary Table 3.** Relative effect sizes of different interventions according to network meta-analysis (Random model)

(A) The league table of the effect of non-pharmaceutical on overall quality of life.

| ID |  |  |  |  |  |  |  |
| --- | --- | --- | --- | --- | --- | --- | --- |
| 0.04 (-0.67,0.76) | CT+APP |  |  |  |  |  |  |
| 0.24 (-0.53,1.01) | 0.20 (-0.68,1.07) | EMS+PA |  |  |  |  |  |
| 0.30 (-0.10,0.71) | 0.26 (-0.33,0.85) | 0.06 (-0.59,0.72) | UC |  |  |  |  |
| 0.30 (-0.14,0.75) | 0.26 (-0.36,0.88) | 0.06 (-0.62,0.74) | 0.00 (-0.19,0.19) | MC |  |  |  |
| 0.41 (-0.13,0.95) | 0.36 (-0.32,1.05) | 0.17 (-0.58,0.91) | 0.10 (-0.26,0.46) | 0.10 (-0.30,0.51) | CE |  |  |
| 0.46 (-0.18,1.10) | 0.41 (-0.36,1.18) | 0.22 (-0.20,0.64) | 0.15 (-0.35,0.65) | 0.15 (-0.38,0.69) | 0.05 (-0.56,0.67) | PA |  |
| 0.67 (-0.10,1.45) | 0.63 (-0.26,1.51) | 0.43 (-0.34,1.20) | 0.37 (-0.29,1.03) | 0.37 (-0.32,1.05) | 0.26 (-0.49,1.02) | 0.21 (-0.44,0.86) | CT+PA |

(B) The league table of the effect of non-pharmaceutical on PCS.

| ABCDE |  |  |  |  |  |  |  |
| --- | --- | --- | --- | --- | --- | --- | --- |
| 0.13 (-1.06,1.31) | PA |  |  |  |  |  |  |
| 0.17 (-1.23,1.58) | 0.05 (-0.92,1.01) | NS+CE |  |  |  |  |  |
| 0.19 (-1.30,1.69) | 0.07 (-0.84,0.97) | 0.02 (-1.30,1.34) | IPA |  |  |  |  |
| 0.28 (-1.01,1.58) | 0.16 (-0.53,0.84) | 0.11 (-0.98,1.20) | 0.09 (-1.05,1.22) | EMS+CE |  |  |  |
| 0.51 (-0.91,1.92) | 0.38 (-0.60,1.36) | 0.34 (-0.90,1.57) | 0.31 (-1.02,1.65) | 0.23 (-0.88,1.34) | NS |  |  |
| 0.59 (-0.89,2.06) | 0.46 (-0.61,1.53) | 0.42 (-0.89,1.72) | 0.39 (-1.01,1.80) | 0.31 (-0.88,1.49) | 0.08 (-1.24,1.40) | APP+Psy |  |
| 0.50 (-0.60,1.61) | 0.38 (-0.05,0.81) | 0.33 (-0.53,1.19) | 0.31 (-0.69,1.31) | 0.22 (-0.45,0.90) | -0.00 (-0.89,0.88) | -0.08 (-1.06,0.89) | UC |

(C) The league table of the effect of non-pharmaceutical on MCS.

| ABCDE |  | |  | |  | |  | |  |  |  |
| --- | --- | --- | --- | --- | --- | --- | --- | --- | --- | --- | --- |
| 0.24 (-0.71,1.19) | | PA | |  | |  | |  | | |  |
| 0.25 (-0.91,1.41) | | 0.01 (-0.78,0.80) | | APP+Psy | |  | |  | | |  |
| 0.45 (-0.58,1.49) | | 0.21 (-0.33,0.76) | | 0.20 (-0.69,1.10) | | EMS+CE | |  | | |  |
| 0.43 (-0.46,1.33) | | 0.19 (-0.11,0.49) | | 0.18 (-0.55,0.92) | | -0.02 (-0.54,0.50) | | UC | | |  |
| 0.50 (-0.64,1.64) | | 0.25 (-0.38,0.89) | | 0.25 (-0.77,1.26) | | 0.04 (-0.80,0.88) | | 0.06 (-0.64,0.77) | | | IPA |


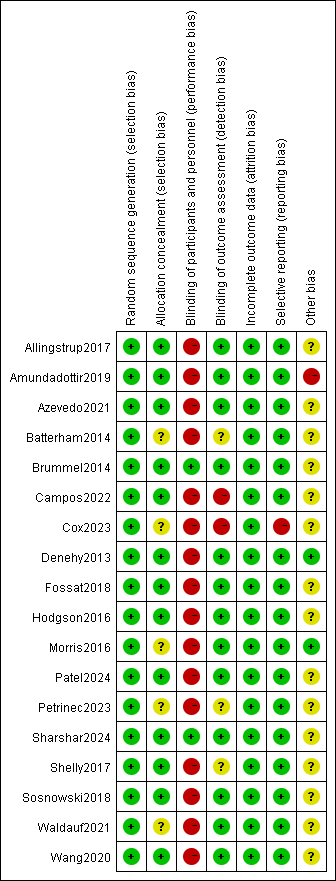


**Supplementary Figure S1.** Quality assessment of included studies using the Cochrane Risk of Bias tool.


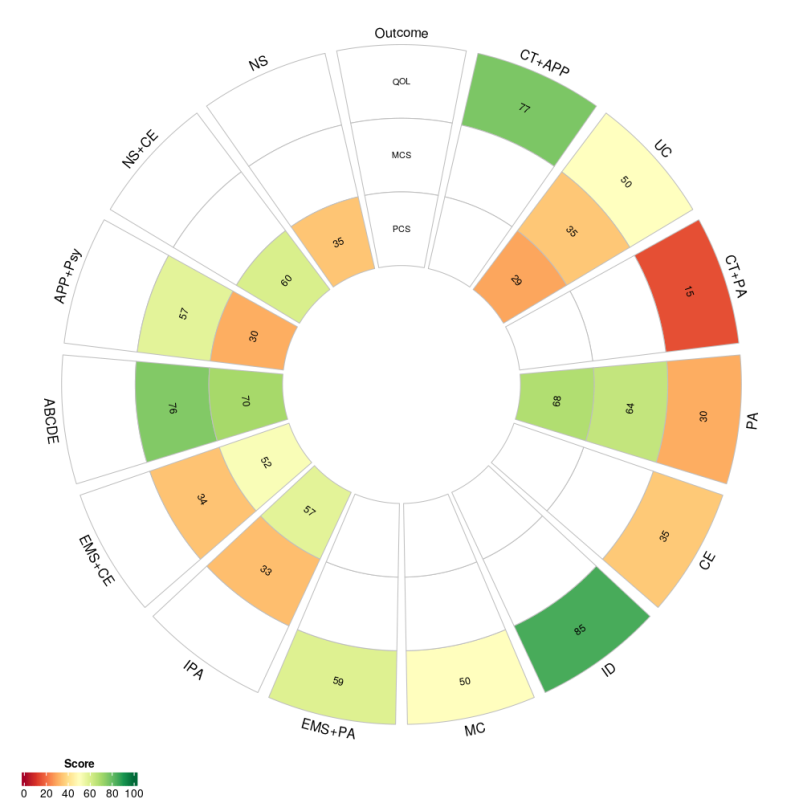


Figure S2. Rank‑heat plot illustrating the comparative performance of non‑pharmacological interventions (NPIs) across three health‑related quality of life (HRQoL) domains: Physical Component Summary (PCS), Mental Component Summary (MCS), and overall Quality of Life (QOL). Each concentric ring represents one HRQoL domain, while each radial segment corresponds to a specific intervention. Color gradients reflect the relative ranking probability or SUCRA‑based performance, with higher values (green) indicating superior effectiveness and lower values (red) indicating poorer performance. This visualization provides an integrated overview of how each intervention performs across multiple HRQoL dimensions, facilitating rapid comparison of multidomain therapeutic effects.


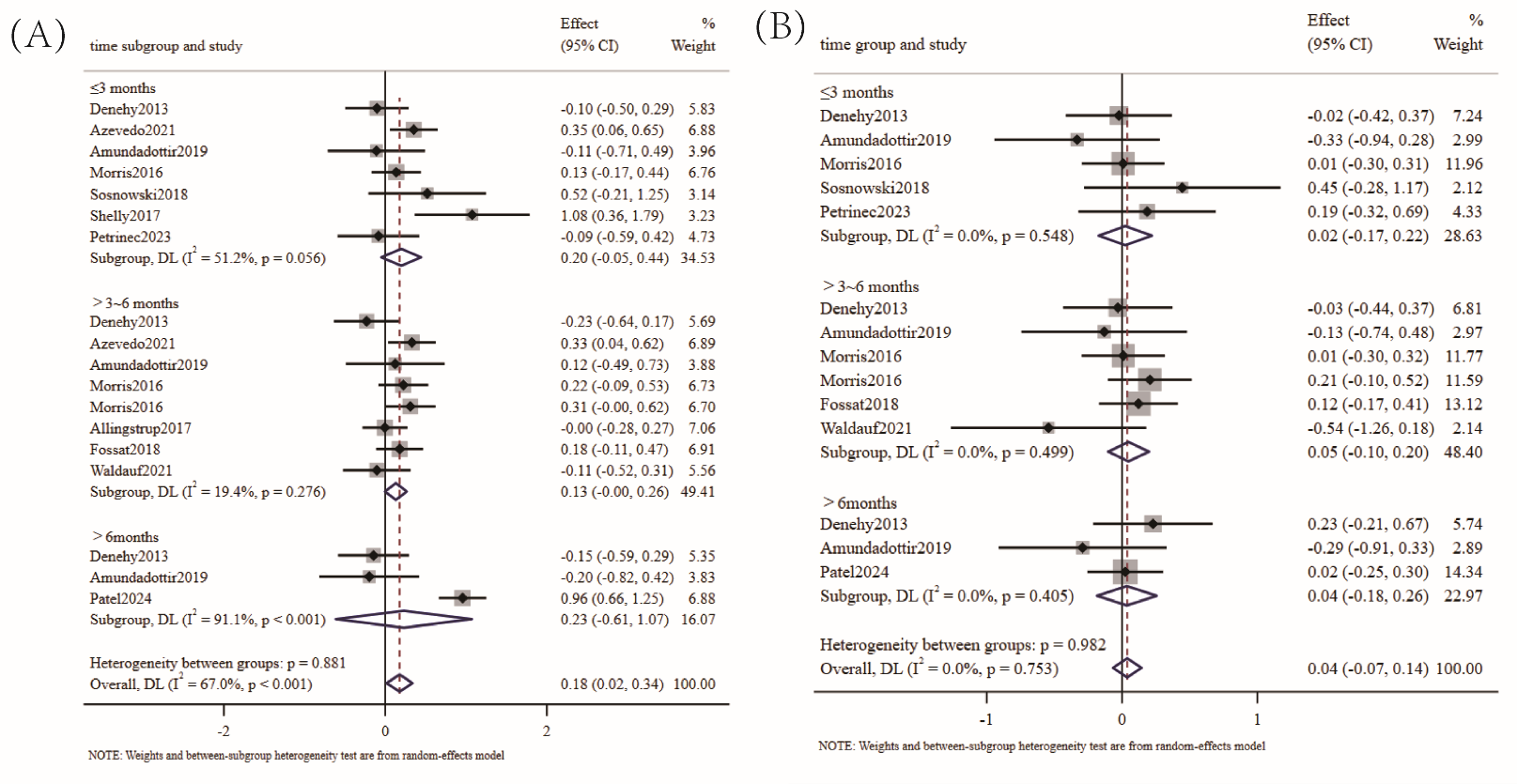


**Supplementary Figure S3.** The result of Subgroup analysis based on the follow-up time. (A) PCS ; (B) MCS

**
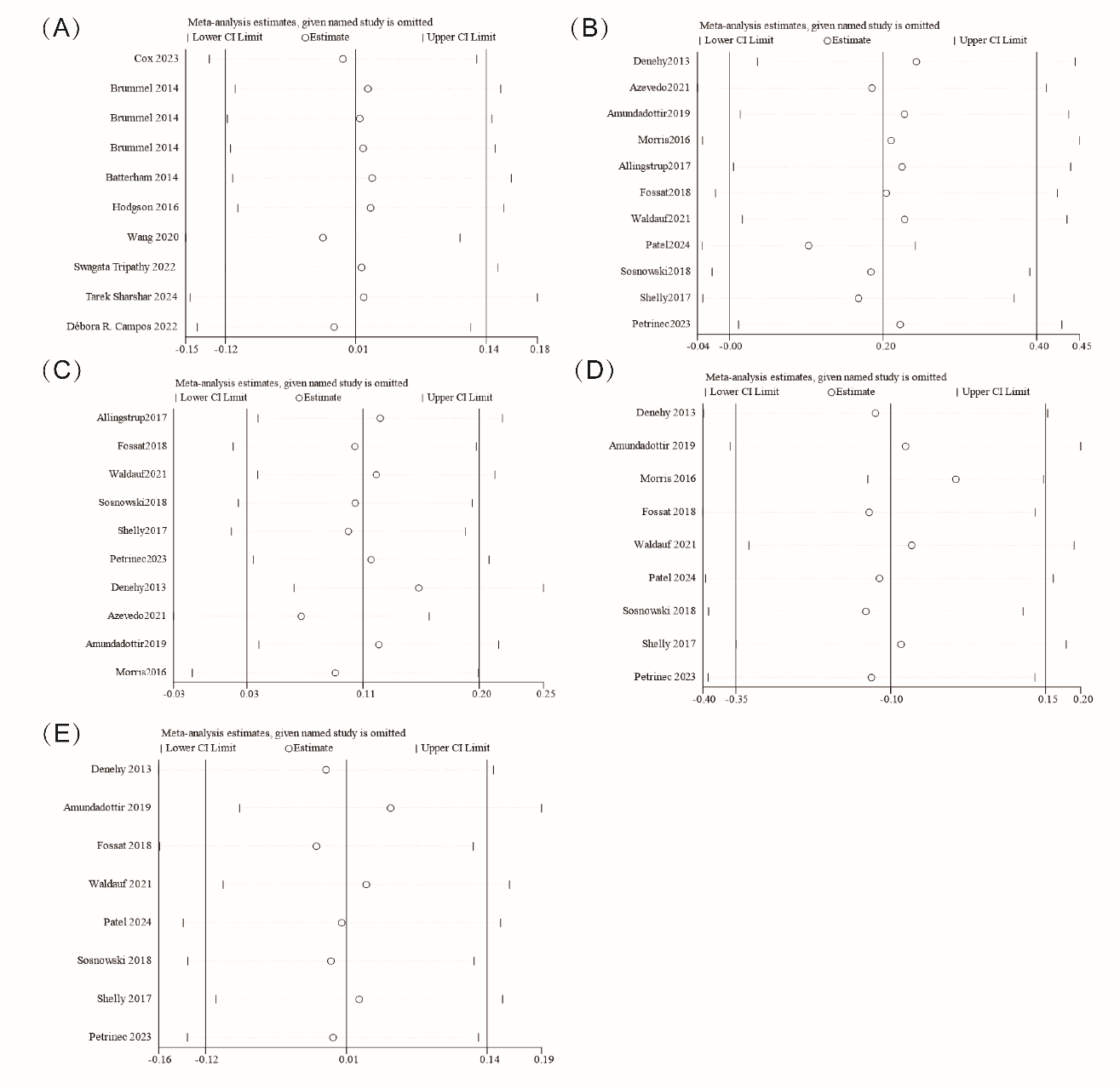
**

**Supplementary Figure S4.** The result of sensitivity analyses.(A) Overall quality of life (B) PCS (C) Sensitivity analysis after excluding the literature in PCS, fixed effect model (D) MCS (E) Sensitivity analysis after excluding the literature in MCS, fixed effect model


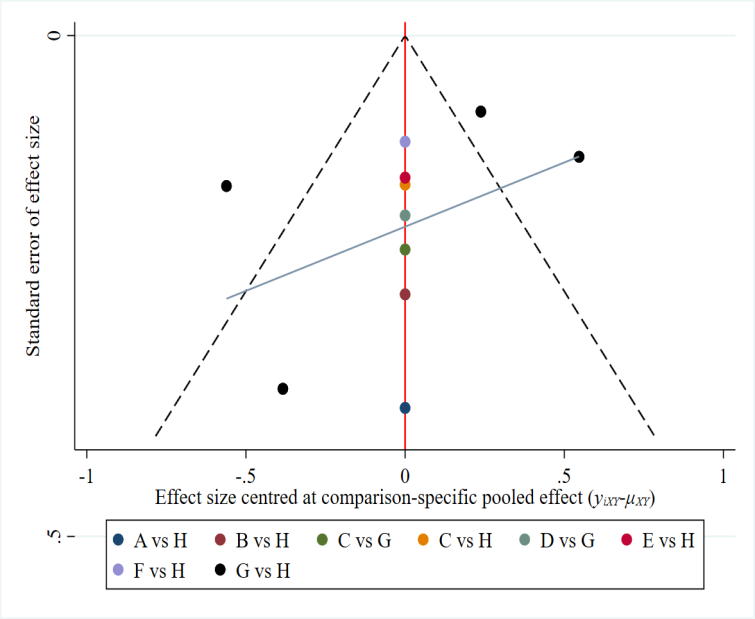


**Supplementary Figure S5.** Funnel plots of trials included in the network meta-analysis on PCS symptoms. SMD: standard mean difference.
